# Revisiting the Awareness and Understanding the Associations between Intracranial Tumors and Optic Neuropathy

**DOI:** 10.3390/diagnostics11122374

**Published:** 2021-12-16

**Authors:** Tomasz Żarnowski, Urszula Łukasik, Iwona Żarnowska, Ewa Kosior-Jarecka

**Affiliations:** 1 Department of Diagnostics and Microsurgery of Glaucoma, Medical University of Lublin, 20-079 Lublin, Poland; urszula.lukasik@wp.pl (U.Ł.); ewakosiorjarecka@umlub.pl (E.K.-J.); 2 Department of Paediatric Neurology, Medical University of Lublin, 20-059 Lublin, Poland; iwonazarnowska@umlub.pl

**Keywords:** optic neuropathy, intracranial tumors, pituitary adenoma, chiasmal syndromes, neuroimaging, cupping, glaucoma

## Abstract

The aim of this paper is to report clinically various cases of intracranial tumors in patients referred to glaucoma clinic for consultation. The secondary aim was to increase the awareness of intracranial tumors in atypical cases of glaucoma. We present the retrospective analysis of five patients referred to glaucoma clinic for consultation. Due to atypical course of the disease, in addition to standard glaucoma examinations, all patients had a neurologic full visual field, color vision, and MRI done. In all patients, intracranial malignancies were found, some patients underwent surgery of the lesions with consecutive clinical improvements. Interestingly, in some patients, coexisting glaucoma was diagnosed. Patients were selected deliberately to present a wide spectrum of possible clinical scenarios when glaucoma may be complicated by intracranial tumors. Sometimes, the relevance of intracranial tumors with respect to their influence on the clinical picture of the optic nerve cannot be established. To conclude, in the “atypical cases of glaucoma” the assessment of the optic nerve may indicate the necessity of neuroimaging in differential diagnostics.

## 1. Introduction

Visual disturbances are frequently presenting symptoms of intracranial tumors. Initial symptoms are transient visual obscurations, visual loss, or visual field (VF) defects [1]. Thus, ophthalmologists are often the first physicians to encounter a patient with clinical manifestations of brain tumors. From an ophthalmologist perspective, a presentation of optic neuropathy may not fit to the picture of any neuropathy seen in everyday clinical practice. In fact, from the beginning of the diagnosis, eye doctors concentrate on the most popular and commonly seen optic neuropathies that include glaucoma, ischemic optic neuropathy, or hereditary optic neuropathies. Nevertheless, rarer neuropathy like compressive neuropathy may not be the first thing to think about. However, the mere fact of practicing in a very busy out-patient glaucoma department does not exclude the possibility of encountering other, less frequent, causes of neuropathy. Contrarily, there is even more chance to diagnose non-glaucomatous neuropathy by a glaucoma specialist than by a comprehensive ophthalmologist. Compressive optic neuropathy is less frequent compared to the abovementioned causes of neuropathy, but requires full neuro-ophthalmological examination, including radiologic brain scans. Indeed, vast majority of patients presenting with glaucoma do not require neuroimaging as the diagnosis is straightforward. In order not to overlook intracranial tumors or other serious conditions, it is very important to perform neuroimaging in atypical cases of presumed glaucoma and other optic neuropathies.

First, Pruett in 1973 noticed that due to low awareness of chiasmal compression as intracranial reason of optic neuropathies, these lesions are prone to “diagnostic delay” [2]. Further observations have been published more contemporarily; we performed neuroimaging in 126 patients with so-called “atypical” NTG, showing that 29 (23%) patients had brain pathologies or different pathologies; 18 (14.2%) of them were found to clinically affect the visual pathway [3]. We confirmed that fast progression of VF or worsening of best corrected visual acuity (BCVA) are the most important signs in prompting magnetic resonance (MR) scans, in contrast to young age and unilateral involvement. Choudhari presented a series of six patients receiving treatment for normal tension glaucoma (NTG) or primary open angle glaucoma (POAG) who were finally found to have optic neuropathy secondary to compression of anterior visual pathways (Choudhari et al., 2011) [4]. Since misdiagnosing is common and successful diagnostic process of all forms of optic neuropathy for comprehensive ophthalmologist is not realistic, Ahmed advocates routine neuroimaging to avoid diagnostic delay [5]. This attitude is not widely accepted due to low yield for detecting intracranial pathology, but also because health care systems are usually unable to carry that sort of burden [6].

The aim of the present study was to characterize the wide range of clinically encountered situations of optic neuropathy associated with intracranial tumors. The secondary aim of the study was to increase the awareness of compressive neuropathy that is not very rare but frequently forgotten or overlooked.

The study conformed to the standards set by the Declaration of Helsinki. All patients consented their participation in the study and presentation of their images in publications.

## 2. Case Presentations

### 2.1. Case 1

**Figure 1:**
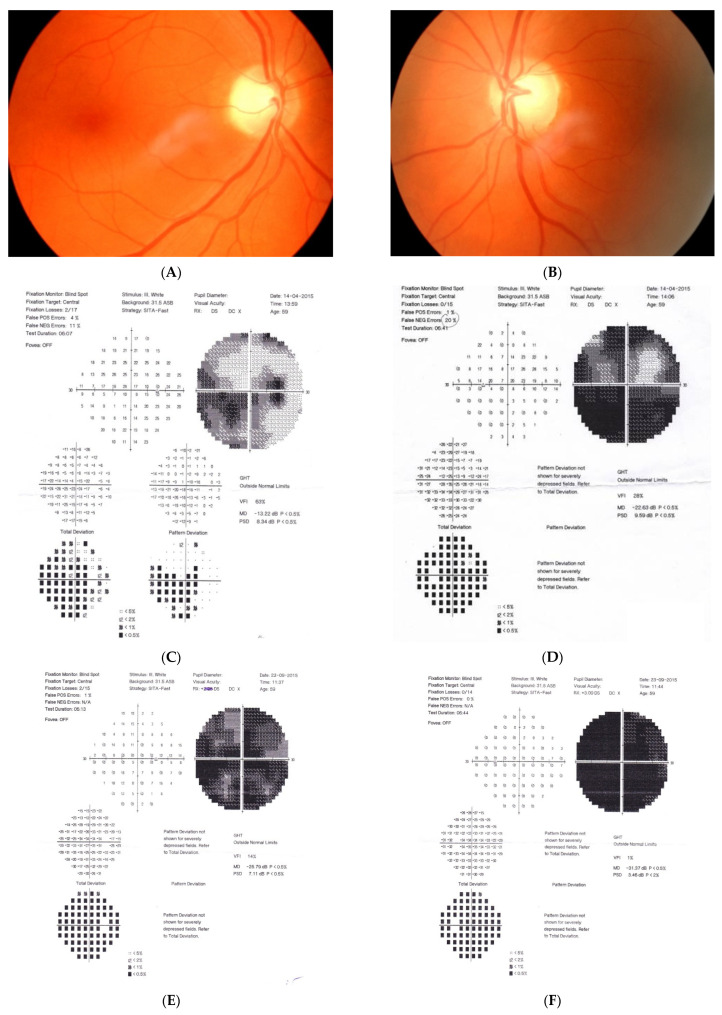

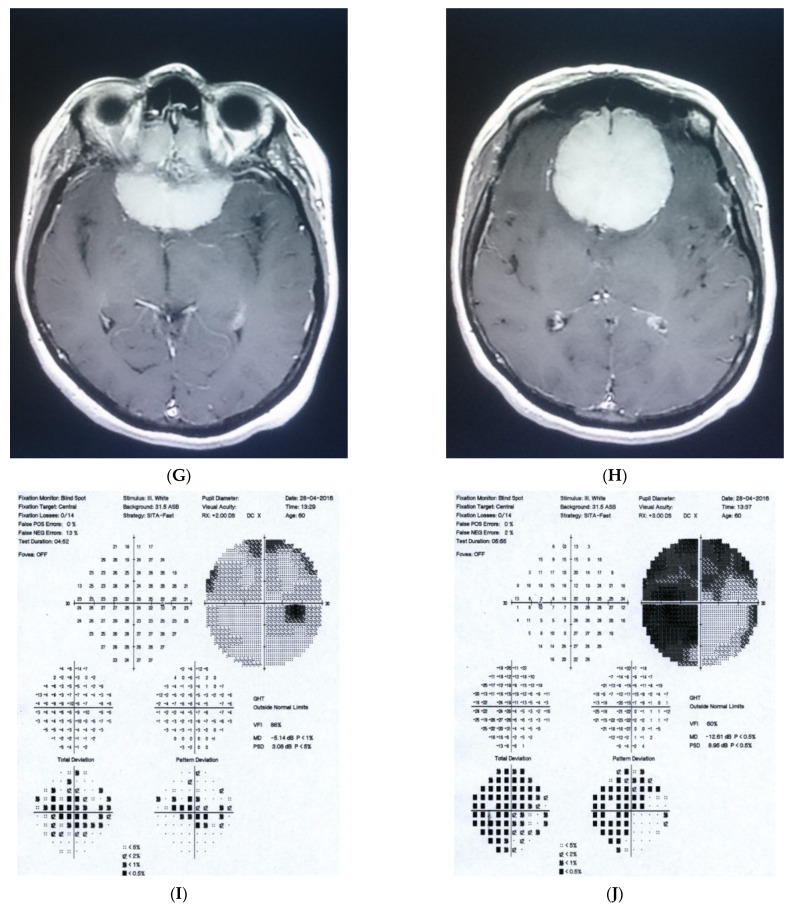
A 60-year-old woman with olfactory groove meningioma. (**A**) Optic disc right eye. (**B**) Optic disc left eye. (**C**) Visual field right eye 04.2015, initial visit. (**D**) Visual field left eye 04.2015, initial visit. (**E**) Visual field right eye 09.2015, showing rapid progression. (**F**) Visual field left eye 09.2015. (**G**,**H**) MRI scans with visible olfactory groove meningioma. (**I**) Visual field right eye 04.2016, after neurosurgical procedure. (**J**) Visual field left eye 04.2016 after neurosurgical procedure.

Figure 1: A 60-year-old female was treated for NTG elsewhere but had been referred to our clinic with deterioration of the visual field. Her BCVA were 0.8 and 0.1, right and left, respectively, and she had normal intraocular pressure (IOP) (19 mmHg). Her parents were both treated for glaucoma, which could account for positive family history (treated with caution as we had not seen the parents). As both optic discs did not look glaucomatous, they looked a little pale but not excavated. The disc appearance did not match very advanced visual fields, so the patients’ drops were discontinued. The patient, seen 3 months later on follow-up visit, exhibited further deterioration of the visual fields with concomitant left vision loss. The BCVA had deteriorated to 0.2 and 0.02, right and left, respectively. An MR scan revealed olfactory groove meningioma that was successfully and subtotally removed by bilateral craniotomy. The BCVA improved to 0.9 and 0.9, right and left eye, respectively, and the VF improved significantly (RE completely, LE—significant loss remained). 

Summary: this is a case of rapid bilateral vision loss with regard to the BCVA and VF (too rapid for glaucoma), in addition, VF did not match optic disc appearance. Positive or “pseudo-positive” family history may be misleading, causing protracted, unnecessary topical treatment, especially in case of putative NTG.

### 2.2. Case 2

**Figure 2:**
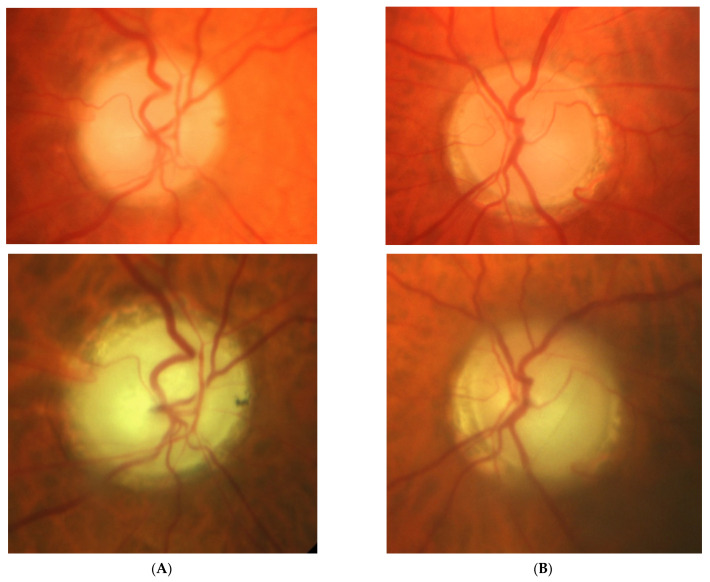

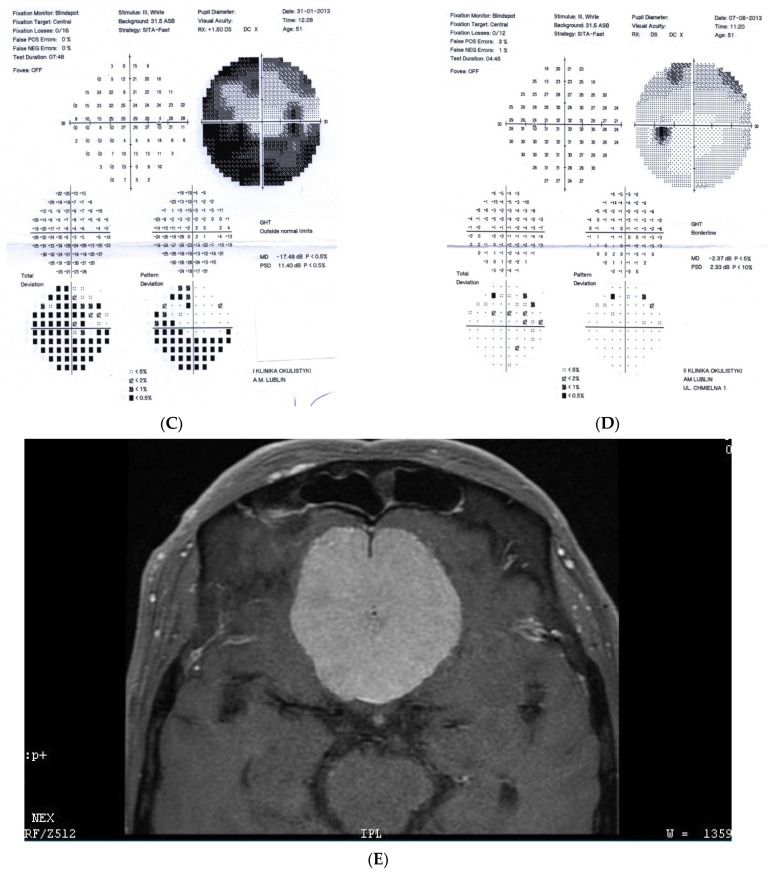

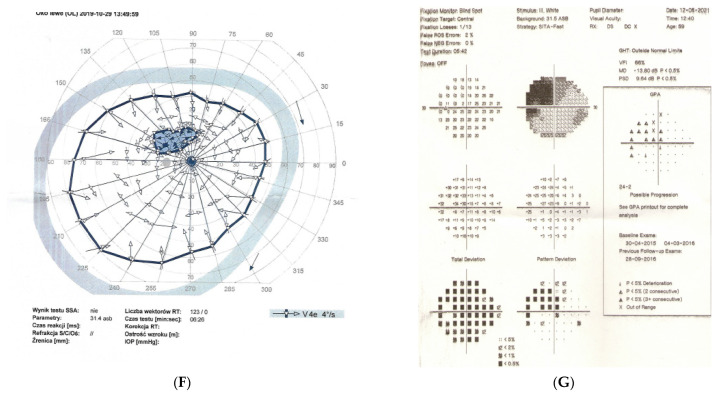
A 56-year-old male with intracranial meningioma. (**A**) Optic disc right eye. (**B**) Optic disc left eye. (**C**) Visual field right eye 01.2013 at the time of trabeculectomy. (**D**) Visual field left eye 01.2013 at the time of trabeculectomy. (**E**) MRI scan with visible intracranial meningioma. (**F**) Semikinetic visual field (SKP) left eye 10.2019 after neurosurgical procedure. (**G**) Visual field left eye 05.2021.

Figure 2: A 56-year-old male treated for POAG with high IOP (30–48 mmHg) for a couple of years but after initial success of drops, he was referred to the clinic due to high pressures (over 40 mmHg). His mother was blind due to glaucoma (confirmed). He underwent trabeculectomy in both eyes when BCVA was 0.5–1.0, but 3 years later, vision deteriorated in both eyes (especially in right eye) despite IOP being maintained around the low teens. VF loss observed over 3 years seemed to be consistent with glaucoma and the island of central vision was lost last. The rapid decrease in central visual acuity in the presence of low and stable IOP was the reason for neuroimaging. He had an MR scan done that revealed an intracranial meningioma that was totally resected by bilateral craniotomy. The right eye is blind and the left eye has some useful VF with BCVA around 0.1 and has been stable for 2 years now. 

Summary: this is true high tension primary glaucoma with a family history that progressed despite successful filtering surgeries. The true family history does not exclude intracranial malignancy, if the course of glaucoma is not typical (long-lasting deterioration after successful IOP drop and atypical pallor of the disc). It is difficult to determine the exact impact of high IOP vs. anterior visual pathway compression on vision loss in this patient. Additionally, disc pallor, a typical sign of compressive neuropathy, may be observed also in juvenile glaucomas or in cases with extremely high values of IOP.

### 2.3. Case 3

**Figure 3:**
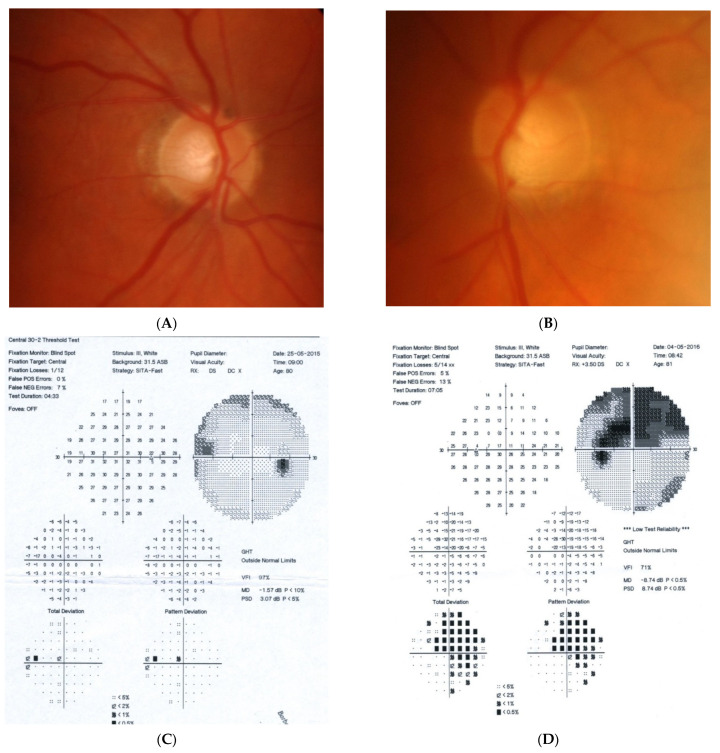
An 82-year-old male with pituitary gland adenoma. (**A**) Optic disc right eye. (**B**) Optic disc left eye. (**C**) Visual field right eye 05.2015 at initial visit. (**D**) Visual field left eye 05.2015 at initial visit.

Figure 3: An 82-year-old male was treated for NTG elsewhere but referred to our clinic for consultation and for left ptosis surgery. His BCVA were 1.0 and 1.0, right and left eye, respectively, and he had normal IOP (14 mmHg). On ophthalmoscopy, both discs look glaucomatous, but the left disc more advanced. Only the left eye exhibited VF changes typical for glaucoma that corresponded ideally with a retinal nerve fiber layer thickness defect in OCT examinations. NTG was stable but unilateral. An MR scan was performed that revealed picture of 4 × 4 mm pituitary microadenoma contacting the chiasm. In three years’ observation, visual field and the tumor size remain stable. 

Summary: this is the case of unilateral stable glaucoma with coexisting pituitary adenoma. It is unclear if the combination of glaucoma and microadenoma is pure coincidence, or if the microadenoma is responsible for the neuropathy.

### 2.4. Case 4 

**Figure 4:**
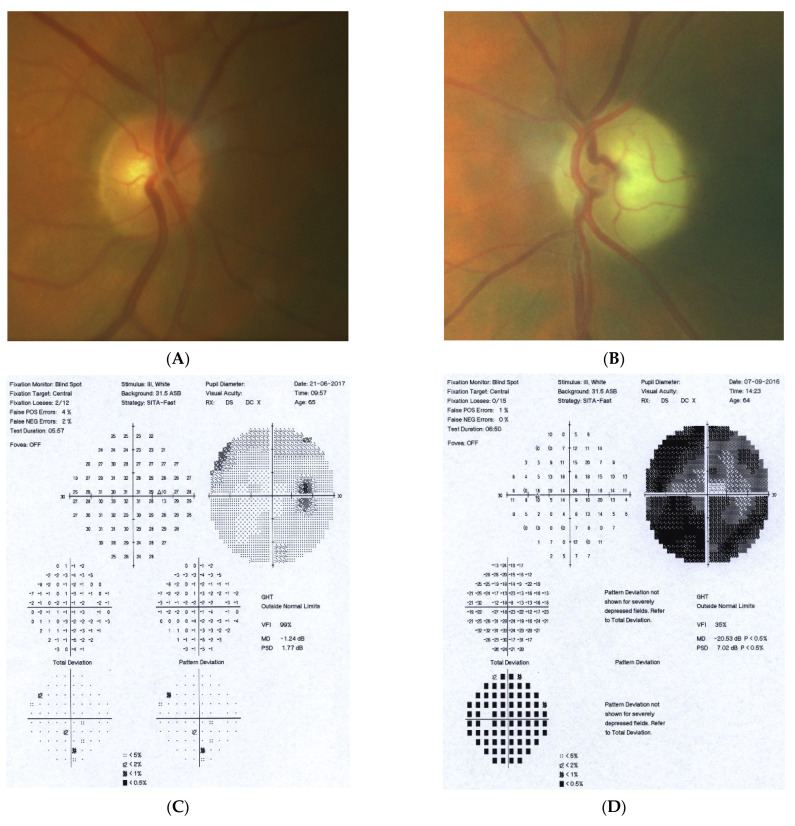

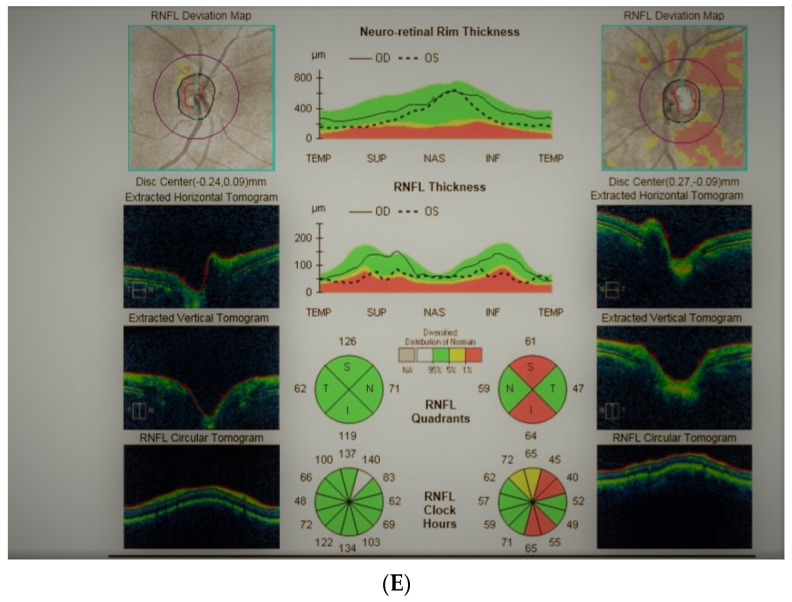
A 65-year-old female with optic disc meningioma. (**A**) Optic disc right eye. (**B**) Optic disc left eye. (**C**) Visual field right eye 06.2017. (**D**) Visual field left eye 06.2017. (**E**) OCT of optic disc with RNFL measurements.

Figure 4: A 65-year-old hyperopic female was referred to our clinic because she developed left eye pallor with consistent VF loss. Her BCVA was 1.0 with correction +4.5 DSph and 0.5+ with correction +4.5 DSph, right and left eye, respectively. The IOP was 15 and 16 mmHg, right and left eye, respectively. Her angle in gonioscopy was narrow (I/II deg.), but neither acute nor prodromal glaucoma were confirmed, which is why an MR was performed. It revealed a left optic nerve sheath meningioma measuring 11 × 12 × 7 mm involving optic nerve canal. The tumor was totally removed by left craniotomy and pathology confirmed a diagnosis of psammomatous meningioma. The patient is stable and continuously observed; the BCVA 2 years after surgery is the same, 1.0 and 0.4, right and left eye, respectively. 

Summary: this straightforward case of unilateral pallor of the optic disc justifies outright MR but, nevertheless, an MR may be retarded by the belief that a unilateral NTG could exist even without excavation, or by the suspicion of acute angle closure in the past. After acute angle closure in the disc, more pallor than cupping may be observed.

### 2.5. Case 5 

**Figure 5:**
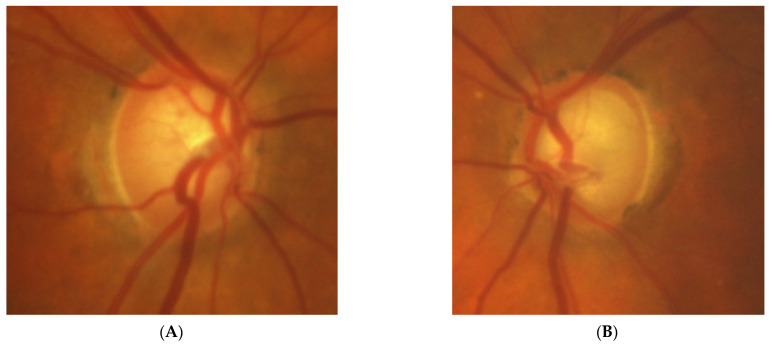

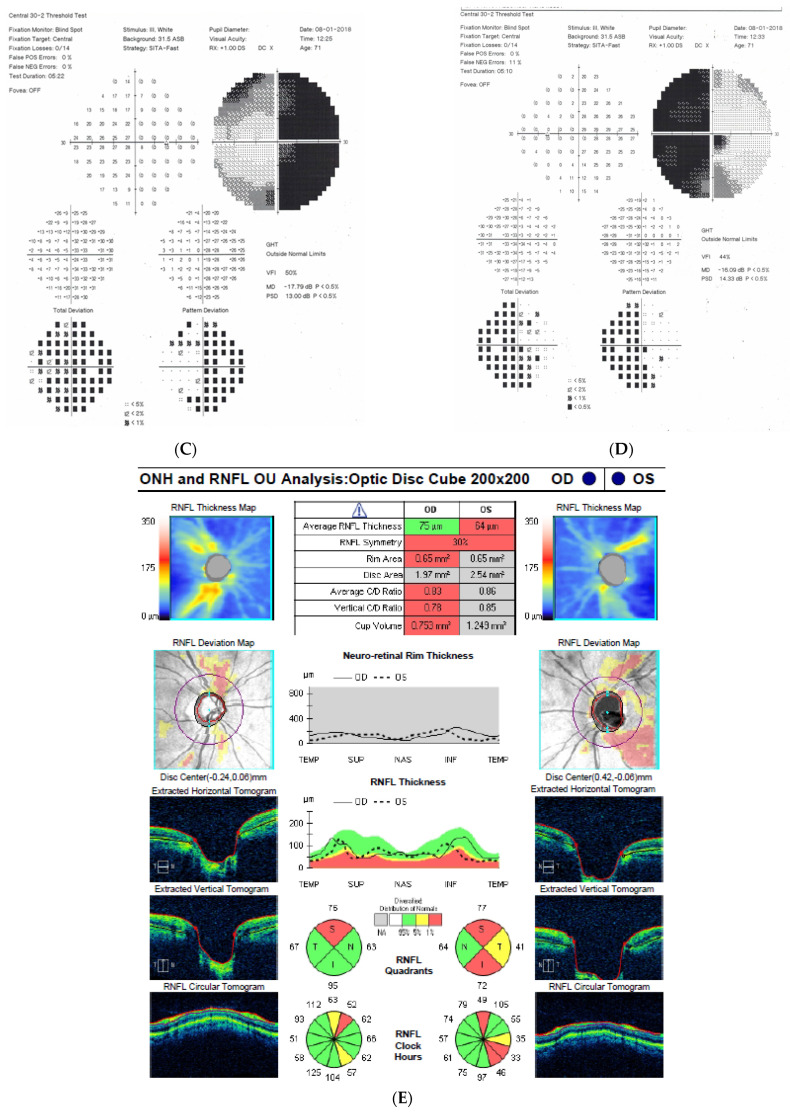

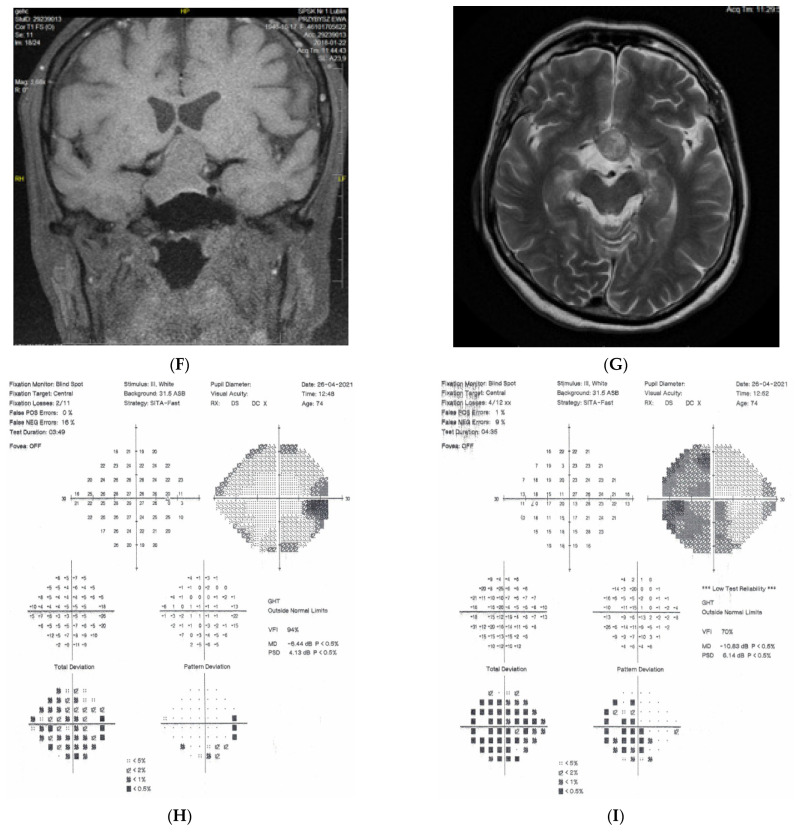
A 70-year-old female with pituitary gland macroadenoma. (**A**) Optic disc right eye. (**B**) Optic disc left eye. (**C**) Visual field right eye 04.2018 at initial visit. (**D**) Visual field left eye 04.2018 at initial visit. (**E**) OCT of optic disc with RNFL measurements 04.2018 at initial visit. (**F**,**G**) MRI scans with visible pituitary macroadenoma. (**H**) Visual field right eye 04.2021 after the neurosurgical intervention. (**I**) Visual field left eye 04.2021 aft.er the neurosurgical intervention.

Figure 5: A 70-year-old female was referred to our clinic because her NTG progressed. Her BCVA was 0.5 and 1.0, right and left eye, respectively. The IOP on glaucoma drops was 15 and 16 mm Hg, right and left eye, respectively. Both discs looked clearly glaucomatous with C/D = 0.8–0.9 with disc hemorrhage on the right side. However, the VF revealed bitemporal hemianopia hiding typical glaucomatous field loss. An MR scan was immediately performed and revealed pituitary macroadenoma (24 × 30 × 20 mm) affecting the chiasm. The tumor was removed by transsphenoidal resection. The VF improved very rapidly after surgery and has remained stable for 4 years. 

Summary: this is a typical case of pituitary macroadenoma affecting the chiasm with progressive VF loss and typical bitemporal hemianopia. Coexistence of true glaucoma is rare; interestingly, the progression of glaucoma was halted after the tumor was excised. The influence of the adenoma on the optic disc appearance is uncertain.

## 3. Discussion

### 3.1. Neuroophthalmologist Perspective

In this study all described tumors were benign, but caused the injury due to mass effect and/or the compression on the visual pathway. There are a number of clinical scenarios of brain tumors affecting the eye.

1. Lesions developing in association with an optic nerve develop unilaterally and are characterized by optic disc pallor, VF defects, and OCT findings (their morphology may differ from classical glaucomatous defects).
2. Chiasmal lesions develop slowly and frequently, bilaterally but asymmetrically. MR scans usually suggest pituitary adenoma, craniopharyngioma, or olfactory groove meningioma of this region. In almost two thirds of the patients with chiasmal syndrome, this is due to pituitary macroadenomas [7]. Virtually all patients have asymmetrical bilateral visual field loss, majority decreased color vision, half decreased visual acuity, and only one-third optic disc pallor [8].
3. It is noteworthy that retrotectal or even retrochiasmal lesions are unlikely to be misdiagnosed as causes of optic neuropathy. Intracranial lesions, if develop rapidly, may evoke intracranial hypertension that will usually be diagnosed promptly as, e.g., papilloedema. If not diagnosed, sooner or later the patient will be referred due to the consequences of raised intracranial pressure or malignancy itself. If the lesion develops slowly, the ophthalmic presentation will be a visual field defect, with respect to the vertical meridian, but pallor does not develop as the lesion is located distal to ganglion cells. In these typical situations when retrotectal areas of visual pathways are involved, a picture of the right or left homonymous hemianopia is present, with normal discs and pupillary reflexes. Such pictures develop in stroke, tumors, and trauma, etc.

### 3.2. Glaucomatologist Perspective

Sometimes, an atypical course of glaucoma led us to perform neuroimaging of the brain. In some 20–25% of the atypical cases of presumed glaucoma, intracranial tumors exist. In fact, cupping of the optic nerve, classically a sign of glaucoma, was demonstrated in 16 out of 250 patients (6.4%) with slowly growing lesions compressing the anterior visual pathway [9]. Optic disc cupping, when concomitant with chiasmal syndrome, almost always retards the diagnosis as it is diagnosed as glaucoma first. There are also other rare causes of optic disc cupping, such as methanol poisoning and ischemic optic neuropathy. Similarly, our observations indicate that chiasmal compression may produce either simple pallor or disc excavation. Regarding the latter one, it is difficult to determine if there is a coincidence or true relationship. There could be myriad of diverse clinical situations: real compressive neuropathy, neuropathy resembling glaucoma, glaucoma enhanced by the fact of existence of slowly growing tumors, glaucoma, and coexisting tumor that is not relevant and, therefore not associated with glaucoma. This determination is not based on the quantitative criteria, but on clinical experience that may vary and is not standardized. The coexistence of a pituitary gland tumor and cupping asks the question of whether the optic disc cupping develops as a result of the glaucomatous process or the slow growth of the tumor causes the glaucoma-mimicking changes. The answer is crucial when assessing the need for antiglaucoma treatment in patients after tumor removal.

Overall, it is strongly suggested to perform at least minimal neuro-ophthalmologic assessment of the optic nerve, including pupil reactions, color vision, visual acuity, visual field examination (usually Goldman neurologic full field), and an OCT exam in cases of atypical glaucoma including normal IOP as one of its signs. In “atypical cases of glaucoma”, the detailed assessment of the optic nerve may also indicate the necessity of neuroimaging in differential diagnostics. These examinations are entirely within the scope of a competent ophthalmologist and could enhance the ability to identify a neurological abnormality, sometimes a serious one. There are additional areas of study that could be intriguing for ophthalmologists is: if there is a coexistence of glaucoma and intracranial malignancy; if there is an influence of intracranial tumors on the progression of glaucoma; and if this is a purely unrelated coexistence or perhaps slowly growing tumors that may resemble glaucomatous optic neuropathy.

## Data Availability

The data are available from the corresponding author after request.
